# Tris(3-methyl­anilinium) tetra­chlorido­zincate chloride hemihydrate

**DOI:** 10.1107/S1600536811044230

**Published:** 2011-10-29

**Authors:** Ming-Liang Liu

**Affiliations:** a College of Chemistry and Chemical Engineering, Southeast University, Nanjing 211189, People’s Republic of China

## Abstract

The asymmetric unit of the title compound, (C_7_H_10_N)_3_[ZnCl_4_]Cl·0.5H_2_O, consists of three 3-methyl­anilinium cations, one tetrahedral tetra­chloridozincate anion and one chloride anion and a water mol­ecule, which lies on a twofold axis. The components are linked into chains parallel to the *a* axis by N—H⋯Cl hydrogen bonds.

## Related literature

For background to ferroelectric metal-organic complexes, see: Zhang *et al.* (2009 ▶, 2010 ▶); Ye *et al.* (2010 ▶). For a related structure, see: Rademeyer *et al.* (2005 ▶).
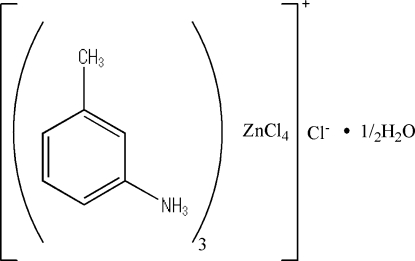


## Experimental

### 

#### Crystal data

(C_7_H_10_N)_3_[ZnCl_4_]Cl·0.5H_2_O
*M* *_r_* = 576.13
Monoclinic, 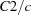

*a* = 26.844 (5) Å
*b* = 7.7071 (15) Å
*c* = 28.605 (6) Å
β = 114.52 (3)°
*V* = 5385 (2) Å^3^
*Z* = 8
Mo *K*α radiation
μ = 1.42 mm^−1^
*T* = 293 K
0.36 × 0.32 × 0.28 mm

#### Data collection

Rigaku Mercury2 diffractometer
Absorption correction: multi-scan (*CrystalClear*; Rigaku, 2005 ▶) *T*_min_ = 0.963, *T*_max_ = 0.971
21123 measured reflections
4728 independent reflections
2941 reflections with *I* > 2σ(*I*)
*R*_int_ = 0.114

#### Refinement

*R*[*F* ^2^ > 2σ(*F* ^2^)] = 0.060
*wR*(*F* ^2^) = 0.135
*S* = 0.99
4728 reflections
286 parameters
1 restraint
H atoms treated by a mixture of independent and constrained refinement
Δρ_max_ = 0.46 e Å^−3^
Δρ_min_ = −0.34 e Å^−3^

### 

Data collection: *CrystalClear* (Rigaku, 2005 ▶); cell refinement: *CrystalClear*; data reduction: *CrystalClear*; program(s) used to solve structure: *SHELXS97* (Sheldrick, 2008 ▶); program(s) used to refine structure: *SHELXL97* (Sheldrick, 2008 ▶); molecular graphics: *SHELXTL* (Sheldrick, 2008 ▶); software used to prepare material for publication: *SHELXTL*.

## Supplementary Material

Crystal structure: contains datablock(s) I, global. DOI: 10.1107/S1600536811044230/go2034sup1.cif

Structure factors: contains datablock(s) I. DOI: 10.1107/S1600536811044230/go2034Isup2.hkl

Additional supplementary materials:  crystallographic information; 3D view; checkCIF report

## Figures and Tables

**Table 1:** Hydrogen-bond geometry (Å, °)

*D*—H⋯*A*	*D*—H	H⋯*A*	*D*⋯*A*	*D*—H⋯*A*
N1—H1*B*⋯Cl2^i^	0.89	2.34	3.176 (4)	157
N2—H2*C*⋯Cl4^ii^	0.89	2.40	3.257 (4)	160
N3—H3*A*⋯Cl6^ii^	0.89	2.34	3.231 (5)	176

Symmetry codes: (i) 
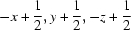
; (ii) 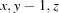.

**Fig. 1.**
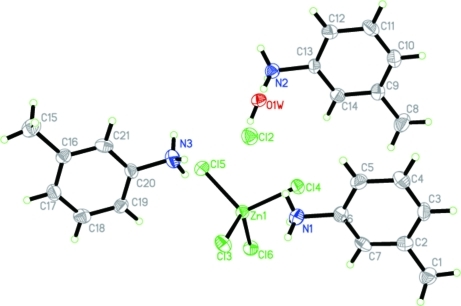
The molecular structure of the title compound, showing the atomic numbering scheme with 30% probability displacement ellipsoids.

**Fig. 2.**
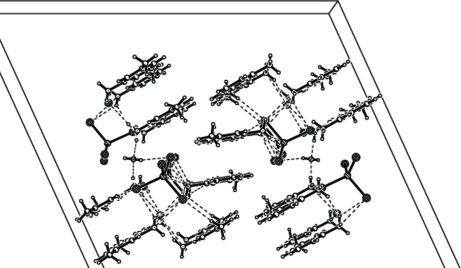
A view of the packing of the title compound, stacking along the b axis. Dashed lines indicate hydrogen bonds.

## supplementary crystallographic information

### Comment

Recently much attention has been devoted to Metal–organic crystals containing organic ions and metal ions due to the tunability of their special structural features and their interesting physical properties (Zhang *et al.*, 2009; Ye *et al.*, 2010; Zhang *et al.*, 2010.). In our laboratory, the title compound has been synthesized and its crystal structure is herein reported.

The molecule of the title compound, [(C_7_H_10_N)_3_(ZnCl_4_)Cl]0.5H_2_O has an asymmetric unit that consists of three C_7_H_10_N cations, one zinc tetrachloride anion and one chloride anion all in general positions and a half water molecule which lies on a twofold axis (Fig 1).The non-hydrgen atoms of C_7_H_10_N cations are nearly coplanar, the zinc tetrachloride anion is a distorted tetrahedron, the average Zn-Cl bond distances range from 2.2526 (14)Å to 2.2898 (16)Å, the Cl-Zn-Cl angles range from 112.49 (6)° to 114.39 (6)°. In the shructure there are some hydrogen bonds (N1-H1B···Cl2, N2-H2C···Cl4, N3-H3A···Cl6) linking the ions of the asymmetric unit. The asymmetric units are linked into chains parallel to *a* axis by N-H···Cl hydrogen bonds (Fig 2, Table 1).

### Experimental

3.21 g (0.03 mol) of 3-methylbenzenamine was firstly dissolved in 30 ml methanol to which 1.1 g (0.03 mol) of hydrochloric acid was added to afford the solution. Then the 1.36 g (0.01 mol) zinc chloride was dissolved in 20 ml methanol and hydrochloric acid and the obtained solution was mixed the above under stirring at the ambient temperature. Single crystals suitable for X-ray structure analysis were obtained by the slow evaporation of the above solution after 4 days in air.

The dielectric constant of the compound as a function of temperature indicates that the permittivity is basically temperature-independent (*ε* = C/(T–T_0_)), suggesting that this compound is not ferroelectric or there may be no distinct phase transition occurring within the measured temperature within the measured temperature (below the melting point).

### Refinement

H atoms were placed in calculated positions (N—H = 0.89 Å; C—H = 0.93Å for C*sp^2^* atoms and C—H = 0.96Å and 0.97Å for C*sp^3^* atoms), assigned fixed *U*_iso_ values [*U*_iso_ = 1.2*U*eq(C*sp^2^*) and 1.5*U*eq(C*sp^3^*,*N*)] and allowed to ride, The H1WA atom bonding with O was found with O—H bond distance of 0.9084Åin the difference electron density map.

### Crystal data

**Table:**

(C_7_H_10_N)_3_[ZnCl_4_]Cl·0.5H_2_O	*F*(000) = 2376
*M**_r_* = 576.13	*D*_x_ = 1.421 Mg m^−^^3^
Monoclinic, *C*2/*c*	Mo *K*α radiation, λ = 0.71073 Å
Hall symbol: -C 2yc	Cell parameters from 21123 reflections
*a* = 26.844 (5) Å	θ = 3.1–27.6°
*b* = 7.7071 (15) Å	µ = 1.42 mm^−^^1^
*c* = 28.605 (6) Å	*T* = 293 K
β = 114.52 (3)°	Block, colorless
*V* = 5385 (2) Å^3^	0.36 × 0.32 × 0.28 mm
*Z* = 8	

### Data collection

**Table:**

Rigaku Mercury2 diffractometer	4728 independent reflections
Radiation source: fine-focus sealed tube	2941 reflections with *I* > 2σ(*I*)
graphite	*R*_int_ = 0.114
Detector resolution: 13.6612 pixels mm^-1^	θ_max_ = 25.0°, θ_min_ = 3.0°
CCD_Profile_fitting scans	*h* = −31→31
Absorption correction: multi-scan (*CrystalClear*; Rigaku, 2005)	*k* = −9→9
*T*_min_ = 0.963, *T*_max_ = 0.971	*l* = −34→34
21123 measured reflections	

### Refinement

**Table:**

Refinement on *F*^2^	Secondary atom site location: difference Fourier map
Least-squares matrix: full	Hydrogen site location: inferred from neighbouring sites
*R*[*F*^2^ > 2σ(*F*^2^)] = 0.060	H atoms treated by a mixture of independent and constrained refinement
*wR*(*F*^2^) = 0.135	*w* = 1/[σ^2^(*F*_o_^2^) + (0.0496*P*)^2^ + 6.8371*P*] where *P* = (*F*_o_^2^ + 2*F*_c_^2^)/3
*S* = 0.99	(Δ/σ)_max_ < 0.001
4728 reflections	Δρ_max_ = 0.46 e Å^−^^3^
286 parameters	Δρ_min_ = −0.34 e Å^−^^3^
1 restraint	Extinction correction: *SHELXL97* (Sheldrick, 2008)
Primary atom site location: structure-invariant direct methods	Extinction coefficient: 0.038 (5)

### Special details

**Table:**

Geometry. All e.s.d.'s (except the e.s.d. in the dihedral angle between two l.s. planes) are estimated using the full covariance matrix. The cell e.s.d.'s are taken into account individually in the estimation of e.s.d.'s in distances, angles and torsion angles; correlations between e.s.d.'s in cell parameters are only used when they are defined by crystal symmetry. An approximate (isotropic) treatment of cell e.s.d.'s is used for estimating e.s.d.'s involving l.s. planes.
Refinement. Refinement of *F*^2^ against ALL reflections. The weighted *R*-factor *wR* and goodness of fit *S* are based on *F*^2^, conventional *R*-factors *R* are based on *F*, with *F* set to zero for negative *F*^2^. The threshold expression of *F*^2^ > σ(*F*^2^) is used only for calculating *R*-factors(gt) *etc*. and is not relevant to the choice of reflections for refinement. *R*-factors based on *F*^2^ are statistically about twice as large as those based on *F*, and *R*- factors based on ALL data will be even larger.

### Fractional atomic coordinates and isotropic or equivalent isotropic displacement parameters (Å^2^)

**Table:**

	*x*	*y*	*z*	*U*_iso_*/*U*_eq_	
Zn1	0.05317 (2)	0.72394 (8)	0.18323 (2)	0.0439 (2)	
Cl2	0.18258 (5)	0.19741 (18)	0.24934 (5)	0.0515 (4)	
Cl3	0.12449 (6)	0.69858 (19)	0.15935 (5)	0.0573 (4)	
Cl4	0.09197 (6)	0.67787 (17)	0.27015 (5)	0.0518 (4)	
Cl5	−0.00605 (6)	0.50710 (18)	0.14357 (5)	0.0554 (4)	
Cl6	0.01561 (6)	0.99087 (17)	0.16803 (5)	0.0538 (4)	
C6	0.24123 (19)	0.6393 (7)	0.3266 (2)	0.0415 (13)	
C13	0.12143 (19)	0.1269 (7)	0.34680 (18)	0.0373 (12)	
C20	0.0782 (2)	0.2332 (7)	0.08050 (19)	0.0415 (13)	
C19	0.0685 (2)	0.3746 (7)	0.0489 (2)	0.0461 (14)	
H19	0.0684	0.4863	0.0612	0.055*	
N1	0.21390 (17)	0.5968 (6)	0.27185 (15)	0.0522 (12)	
H1A	0.2061	0.4841	0.2682	0.078*	
H1B	0.2359	0.6226	0.2566	0.078*	
H1C	0.1831	0.6580	0.2574	0.078*	
C14	0.1301 (2)	0.2960 (7)	0.3638 (2)	0.0445 (13)	
H14	0.1179	0.3863	0.3401	0.053*	
N2	0.09265 (15)	0.0929 (5)	0.29173 (15)	0.0441 (11)	
H2A	0.1139	0.1228	0.2761	0.066*	
H2B	0.0619	0.1548	0.2789	0.066*	
H2C	0.0846	−0.0195	0.2867	0.066*	
C4	0.2855 (2)	0.5520 (8)	0.4130 (2)	0.0619 (17)	
H4	0.2970	0.4652	0.4378	0.074*	
C17	0.0593 (2)	0.1800 (7)	−0.0192 (2)	0.0509 (15)	
H17	0.0529	0.1634	−0.0535	0.061*	
N3	0.08751 (19)	0.2594 (6)	0.13476 (16)	0.0588 (13)	
H3A	0.0663	0.1871	0.1427	0.088*	
H3B	0.0793	0.3684	0.1392	0.088*	
H3C	0.1225	0.2386	0.1551	0.088*	
C21	0.07792 (19)	0.0668 (7)	0.0634 (2)	0.0455 (14)	
H21	0.0839	−0.0262	0.0858	0.055*	
C7	0.2497 (2)	0.8116 (7)	0.3409 (2)	0.0472 (14)	
H7	0.2368	0.8979	0.3160	0.057*	
C11	0.1671 (2)	0.0271 (8)	0.4321 (2)	0.0580 (16)	
H11	0.1798	−0.0637	0.4555	0.070*	
C12	0.1394 (2)	−0.0089 (7)	0.3806 (2)	0.0535 (15)	
H12	0.1331	−0.1230	0.3690	0.064*	
C5	0.2591 (2)	0.5088 (7)	0.3621 (2)	0.0536 (15)	
H5	0.2533	0.3932	0.3520	0.064*	
C16	0.0688 (2)	0.0374 (7)	0.0126 (2)	0.0437 (13)	
C10	0.1761 (2)	0.1962 (8)	0.4495 (2)	0.0539 (15)	
H10	0.1953	0.2179	0.4844	0.065*	
C18	0.0590 (2)	0.3457 (7)	−0.0016 (2)	0.0525 (15)	
H18	0.0524	0.4391	−0.0240	0.063*	
C2	0.2773 (2)	0.8566 (7)	0.3922 (2)	0.0451 (13)	
C3	0.2953 (2)	0.7245 (7)	0.4278 (2)	0.0524 (15)	
H3	0.3145	0.7516	0.4624	0.063*	
C9	0.1570 (2)	0.3331 (7)	0.4158 (2)	0.0474 (14)	
C8	0.1640 (3)	0.5198 (7)	0.4342 (2)	0.0713 (19)	
H8A	0.1715	0.5915	0.4104	0.107*	
H8B	0.1940	0.5273	0.4675	0.107*	
H8C	0.1310	0.5587	0.4363	0.107*	
C1	0.2877 (2)	1.0442 (7)	0.4078 (2)	0.0629 (17)	
H1D	0.3060	1.0990	0.3891	0.094*	
H1E	0.3103	1.0516	0.4440	0.094*	
H1F	0.2535	1.1016	0.4002	0.094*	
C15	0.0684 (2)	−0.1441 (7)	−0.0068 (2)	0.0661 (18)	
H15A	0.0415	−0.2119	−0.0010	0.099*	
H15B	0.1038	−0.1955	0.0112	0.099*	
H15C	0.0596	−0.1408	−0.0429	0.099*	
O1W	0.0000	0.3163 (7)	0.2500	0.0475 (13)	
H1WA	0.003 (3)	0.389 (6)	0.2295 (19)	0.09 (2)*	

### Atomic displacement parameters (Å^2^)

**Table:**

	*U*^11^	*U*^22^	*U*^33^	*U*^12^	*U*^13^	*U*^23^
Zn1	0.0477 (4)	0.0419 (4)	0.0403 (4)	0.0014 (3)	0.0166 (3)	0.0002 (3)
Cl2	0.0475 (8)	0.0584 (9)	0.0469 (8)	−0.0016 (7)	0.0178 (7)	0.0010 (7)
Cl3	0.0543 (9)	0.0729 (10)	0.0506 (9)	0.0074 (8)	0.0275 (8)	0.0081 (7)
Cl4	0.0631 (9)	0.0503 (9)	0.0382 (8)	0.0001 (7)	0.0172 (7)	0.0030 (6)
Cl5	0.0550 (9)	0.0537 (9)	0.0533 (9)	−0.0112 (7)	0.0183 (8)	−0.0056 (7)
Cl6	0.0585 (9)	0.0435 (8)	0.0567 (9)	0.0086 (7)	0.0212 (8)	0.0034 (7)
C6	0.029 (3)	0.047 (3)	0.044 (3)	0.000 (2)	0.012 (3)	0.000 (3)
C13	0.034 (3)	0.046 (3)	0.031 (3)	−0.003 (2)	0.013 (2)	−0.003 (3)
C20	0.041 (3)	0.048 (3)	0.032 (3)	0.000 (3)	0.012 (2)	0.002 (3)
C19	0.050 (3)	0.039 (3)	0.048 (3)	−0.002 (3)	0.019 (3)	−0.005 (3)
N1	0.050 (3)	0.055 (3)	0.044 (3)	−0.012 (2)	0.012 (2)	−0.004 (2)
C14	0.045 (3)	0.043 (3)	0.045 (3)	0.005 (3)	0.018 (3)	0.004 (3)
N2	0.042 (3)	0.044 (3)	0.044 (3)	0.000 (2)	0.014 (2)	−0.001 (2)
C4	0.066 (4)	0.061 (4)	0.047 (4)	0.007 (3)	0.011 (3)	0.017 (3)
C17	0.052 (4)	0.062 (4)	0.042 (3)	−0.006 (3)	0.023 (3)	−0.005 (3)
N3	0.060 (3)	0.069 (3)	0.041 (3)	0.006 (3)	0.014 (2)	0.002 (2)
C21	0.039 (3)	0.042 (3)	0.056 (4)	0.009 (3)	0.020 (3)	0.015 (3)
C7	0.053 (3)	0.046 (4)	0.044 (3)	0.011 (3)	0.022 (3)	0.018 (3)
C11	0.064 (4)	0.060 (4)	0.046 (4)	0.006 (3)	0.019 (3)	0.016 (3)
C12	0.069 (4)	0.040 (3)	0.052 (4)	0.000 (3)	0.026 (3)	0.002 (3)
C5	0.058 (4)	0.040 (3)	0.055 (4)	−0.003 (3)	0.016 (3)	0.002 (3)
C16	0.037 (3)	0.044 (3)	0.053 (4)	−0.007 (2)	0.021 (3)	−0.006 (3)
C10	0.049 (4)	0.070 (4)	0.041 (3)	−0.005 (3)	0.017 (3)	−0.005 (3)
C18	0.050 (4)	0.047 (4)	0.051 (4)	0.003 (3)	0.011 (3)	0.015 (3)
C2	0.039 (3)	0.047 (3)	0.055 (4)	0.002 (3)	0.025 (3)	0.002 (3)
C3	0.054 (4)	0.059 (4)	0.034 (3)	−0.003 (3)	0.008 (3)	−0.007 (3)
C9	0.042 (3)	0.056 (4)	0.044 (3)	0.001 (3)	0.017 (3)	−0.005 (3)
C8	0.083 (5)	0.062 (4)	0.067 (4)	−0.011 (3)	0.029 (4)	−0.022 (3)
C1	0.075 (4)	0.050 (4)	0.067 (4)	−0.005 (3)	0.032 (4)	−0.009 (3)
C15	0.066 (4)	0.054 (4)	0.090 (5)	−0.002 (3)	0.044 (4)	−0.014 (3)
O1W	0.051 (3)	0.039 (3)	0.055 (4)	0.000	0.024 (3)	0.000

### Geometric parameters (Å, °)

**Table:**

Zn1—Cl6	2.2526 (14)	N3—H3B	0.8900
Zn1—Cl5	2.2607 (15)	N3—H3C	0.8900
Zn1—Cl4	2.2898 (16)	C21—C16	1.387 (7)
Zn1—Cl3	2.2924 (16)	C21—H21	0.9300
C6—C5	1.368 (7)	C7—C2	1.385 (7)
C6—C7	1.380 (7)	C7—H7	0.9300
C6—N1	1.464 (6)	C11—C12	1.375 (7)
C13—C12	1.370 (7)	C11—C10	1.380 (7)
C13—C14	1.376 (7)	C11—H11	0.9300
C13—N2	1.462 (6)	C12—H12	0.9300
C20—C19	1.370 (7)	C5—H5	0.9300
C20—C21	1.372 (7)	C16—C15	1.503 (7)
C20—N3	1.480 (6)	C10—C9	1.376 (7)
C19—C18	1.379 (7)	C10—H10	0.9300
C19—H19	0.9300	C18—H18	0.9300
N1—H1A	0.8900	C2—C3	1.378 (7)
N1—H1B	0.8900	C2—C1	1.504 (7)
N1—H1C	0.8900	C3—H3	0.9300
C14—C9	1.389 (7)	C9—C8	1.516 (7)
C14—H14	0.9300	C8—H8A	0.9600
N2—H2A	0.8900	C8—H8B	0.9600
N2—H2B	0.8900	C8—H8C	0.9600
N2—H2C	0.8900	C1—H1D	0.9600
C4—C5	1.370 (7)	C1—H1E	0.9600
C4—C3	1.386 (7)	C1—H1F	0.9600
C4—H4	0.9300	C15—H15A	0.9600
C17—C18	1.373 (7)	C15—H15B	0.9600
C17—C16	1.383 (7)	C15—H15C	0.9600
C17—H17	0.9300	O1W—H1WA	0.843 (10)
N3—H3A	0.8900		
			
Cl6—Zn1—Cl5	114.39 (6)	C6—C7—C2	120.4 (5)
Cl6—Zn1—Cl4	108.46 (6)	C6—C7—H7	119.8
Cl5—Zn1—Cl4	109.79 (6)	C2—C7—H7	119.8
Cl6—Zn1—Cl3	112.49 (6)	C12—C11—C10	120.8 (5)
Cl5—Zn1—Cl3	106.73 (6)	C12—C11—H11	119.6
Cl4—Zn1—Cl3	104.52 (6)	C10—C11—H11	119.6
C5—C6—C7	121.5 (5)	C13—C12—C11	118.5 (5)
C5—C6—N1	119.7 (5)	C13—C12—H12	120.7
C7—C6—N1	118.7 (5)	C11—C12—H12	120.7
C12—C13—C14	121.1 (5)	C6—C5—C4	118.6 (5)
C12—C13—N2	119.9 (5)	C6—C5—H5	120.7
C14—C13—N2	119.0 (4)	C4—C5—H5	120.7
C19—C20—C21	122.5 (5)	C17—C16—C21	117.7 (5)
C19—C20—N3	119.1 (5)	C17—C16—C15	121.7 (5)
C21—C20—N3	118.4 (5)	C21—C16—C15	120.6 (5)
C20—C19—C18	117.7 (5)	C9—C10—C11	120.9 (5)
C20—C19—H19	121.2	C9—C10—H10	119.6
C18—C19—H19	121.2	C11—C10—H10	119.6
C6—N1—H1A	109.5	C17—C18—C19	120.5 (5)
C6—N1—H1B	109.5	C17—C18—H18	119.7
H1A—N1—H1B	109.5	C19—C18—H18	119.7
C6—N1—H1C	109.5	C3—C2—C7	117.8 (5)
H1A—N1—H1C	109.5	C3—C2—C1	121.7 (5)
H1B—N1—H1C	109.5	C7—C2—C1	120.4 (5)
C13—C14—C9	120.6 (5)	C2—C3—C4	121.3 (5)
C13—C14—H14	119.7	C2—C3—H3	119.3
C9—C14—H14	119.7	C4—C3—H3	119.3
C13—N2—H2A	109.5	C10—C9—C14	118.0 (5)
C13—N2—H2B	109.5	C10—C9—C8	121.9 (5)
H2A—N2—H2B	109.5	C14—C9—C8	120.1 (5)
C13—N2—H2C	109.5	C9—C8—H8A	109.5
H2A—N2—H2C	109.5	C9—C8—H8B	109.5
H2B—N2—H2C	109.5	H8A—C8—H8B	109.5
C5—C4—C3	120.4 (5)	C9—C8—H8C	109.5
C5—C4—H4	119.8	H8A—C8—H8C	109.5
C3—C4—H4	119.8	H8B—C8—H8C	109.5
C18—C17—C16	121.7 (5)	C2—C1—H1D	109.5
C18—C17—H17	119.2	C2—C1—H1E	109.5
C16—C17—H17	119.2	H1D—C1—H1E	109.5
C20—N3—H3A	109.5	C2—C1—H1F	109.5
C20—N3—H3B	109.5	H1D—C1—H1F	109.5
H3A—N3—H3B	109.5	H1E—C1—H1F	109.5
C20—N3—H3C	109.5	C16—C15—H15A	109.5
H3A—N3—H3C	109.5	C16—C15—H15B	109.5
H3B—N3—H3C	109.5	H15A—C15—H15B	109.5
C20—C21—C16	119.9 (5)	C16—C15—H15C	109.5
C20—C21—H21	120.0	H15A—C15—H15C	109.5
C16—C21—H21	120.0	H15B—C15—H15C	109.5
			
C21—C20—C19—C18	−0.8 (8)	C18—C17—C16—C15	179.3 (5)
N3—C20—C19—C18	−178.9 (5)	C20—C21—C16—C17	−1.0 (8)
C12—C13—C14—C9	−0.7 (8)	C20—C21—C16—C15	−180.0 (5)
N2—C13—C14—C9	179.6 (4)	C12—C11—C10—C9	0.9 (9)
C19—C20—C21—C16	1.3 (8)	C16—C17—C18—C19	0.2 (8)
N3—C20—C21—C16	179.4 (4)	C20—C19—C18—C17	0.1 (8)
C5—C6—C7—C2	0.8 (8)	C6—C7—C2—C3	−0.5 (8)
N1—C6—C7—C2	−177.4 (4)	C6—C7—C2—C1	178.6 (5)
C14—C13—C12—C11	−0.7 (8)	C7—C2—C3—C4	−0.9 (8)
N2—C13—C12—C11	179.1 (4)	C1—C2—C3—C4	−180.0 (5)
C10—C11—C12—C13	0.5 (8)	C5—C4—C3—C2	2.2 (9)
C7—C6—C5—C4	0.4 (8)	C11—C10—C9—C14	−2.2 (8)
N1—C6—C5—C4	178.6 (5)	C11—C10—C9—C8	177.0 (5)
C3—C4—C5—C6	−1.9 (9)	C13—C14—C9—C10	2.1 (8)
C18—C17—C16—C21	0.3 (8)	C13—C14—C9—C8	−177.2 (5)

### Hydrogen-bond geometry (Å, °)

**Table:**

*D*—H···*A*	*D*—H	H···*A*	*D*···*A*	*D*—H···*A*
N1—H1B···Cl2^i^	0.89	2.34	3.176 (4)	157.
N2—H2C···Cl4^ii^	0.89	2.40	3.257 (4)	160.
N3—H3A···Cl6^ii^	0.89	2.34	3.231 (5)	176.

Symmetry codes: (i) −*x*+1/2, *y*+1/2, −*z*+1/2; (ii) *x*, *y*−1, *z*.
